# Optimizing atrio‐ventricular delay in pacemakers using potentially implantable physiological biomarkers

**DOI:** 10.1111/pace.14434

**Published:** 2022-01-28

**Authors:** Daniel Keene, Alejandra A Miyazawa, Monika Johal, Ahran D Arnold, Nadine Ali, Khulat A Saqi, Katherine March, Leah Burden, Darrel P Francis, Zachary I Whinnett, Matthew J Shun‐Shin

**Affiliations:** ^1^ National Heart and Lung Institute, Imperial College London, Hammersmith Hospital London UK; ^2^ Imperial College Healthcare NHS Trust, Hammersmith Hospital London UK

**Keywords:** atrioventricular delay, haemodynamics, laser Doppler perfusion monitoring, optimization, pacemaker

## Abstract

**Background:**

Hemodynamically optimal atrioventricular (AV) delay can be derived by echocardiography or beat‐by‐beat blood pressure (BP) measurements, but analysis is labor intensive. Laser Doppler perfusion monitoring measures blood flow and can be incorporated into future implantable cardiac devices.

We assess whether laser Doppler can be used instead of BP to optimize AV delay.

**Methods:**

Fifty eight patients underwent 94 AV delay optimizations with biventricular or His‐bundle pacing using laser Doppler and simultaneous noninvasive beat‐by‐beat BP. Optimal AV delay was defined using a curve of hemodynamic response to switching from AAI (reference state) to DDD (test state) at several AV delays (40–320 ms), with automatic quality control checking precision of the optimum.

Five subsequent patients underwent an extended protocol to test the impact of greater numbers of alternations on optimization quality.

**Results:**

55/94 optimizations passed quality control resulting in an optimal AV delay on laser Doppler similar to that derived by BP (median absolute deviation 12 ms).

An extended protocol with increasing number of replicates consistently improved quality and reduced disagreement between laser Doppler and BP optima. With only five replicates, no optimization passed quality control, and the median absolute deviation would be 29 ms. These improved progressively until at 50 replicates, all optimizations passed quality control and the median absolute deviation was only 13 ms.

**Conclusions:**

Laser Doppler perfusion produces hemodynamic optima equivalent to BP. Quality control can be automatic. Adding more replicates, consistently improves quality. Future implantable devices could use such methods to dynamically and reliably optimize AV delays.

**Abbreviations**
AV: atrioventricular
BP: blood pressure
CRT: cardiac resynchronization therapy
SE_OPT_
: Standard error of the optimum.

## 1 INTRODUCTION

Cardiac resynchronization therapy (CRT) improves hemodynamic function, cardiac output and importantly reduces hospitalizations and mortality in eligible patients.^1^, ^2^, ^3^ The devices improve both interventricular synchrony (from which the term “cardiac resynchronization” derives) and atrio‐ventricular (AV) timing.^4^, ^5^, ^6^ The greatest hemodynamic benefit is thought to arise from the time to first ventricular activation.^7^ AV delay needs to maximize left ventricular filling by trading off truncation of the A wave if it is short against truncation of the E wave if it is long. Programming longer AV delays may result in fusion between intrinsic activation and LV pacing, which has the potential to deliver more effective ventricular resynchronization. There are therefore two potential mechanisms of benefit with adjustment of AV delay: optimization of left ventricular filling and more effective ventricular resynchronization.

It is difficult to predict from first principles what the best trade‐off is between these factors in an individual patient. A potential advantage of using a measure of perfusion, rather than electrical measurements, is that it assesses the overall impact on cardiac function. A variety of proxies of cardiac function can be used for this hemodynamic optimization: invasive or noninvasive beat‐by‐beat blood pressure (BP), echocardiography or myocardial contractility.^6^, ^7^, ^8^, ^9^, ^10^, ^11^, ^12^, ^13^, ^14^, ^15^, ^16^, ^17^ None of these methods, however, are feasible for incorporation into a CRT device. Furthermore, optimizations are currently performed in cardiac investigation units by trained members of staff providing only a snapshot in time of the optimal pacemaker settings.

Miniaturized technology is now available to measure blood flow (rather than pressure) using laser Doppler perfusion monitoring. Laser Doppler has been validated in patients with burns, for assessment of viability of skin grafts and guiding therapy in various rheumatological and dermatological conditions.^18^, ^19^ We have previously found that it is sensitive to the hemodynamic changes associated with ventricular arrhythmias.^20^

A key necessity for *any* automated optimization technique is a good method for automatically detecting data that has been disrupted by noise, so that it is not used by the device to apply an incorrect setting. Such noise can arise from spontaneous biological variability, inherent measurement errors as well as patient factors such as movement and respiration.^21^

In this study we test (i) laser Doppler perfusion sensors and their ability to hemodynamically optimize AV delays, (ii) an automated algorithm for deriving the optimum, (iii) an automated algorithm for assessing data quality. The purpose is to determine whether a system with these elements (and therefore completely implantable and automatic) is equivalent to a manual process of hemodynamic optimization using BP.

## 2 METHODS

### 2.1 Study population

The study population included patients from two randomized controlled trials (BRAVO of Biventricular pacing and HOPE‐HF of His bundle pacing) in which patients who received either standard biventricular pacing (pacing using both a right ventricular and a coronary sinus lead) or his bundle pacing underwent AV delay optimization using noninvasive BP. In these studies, our site (Imperial College London) was the core lab for hemodynamic optimization. During the optimization, data from a laser Doppler perfusion sensor applied to the skin was also recorded for later analysis. The inclusion and exclusion criteria from these trials are shown in Table S1.^22^, ^23^

### 2.2 Atrioventricular delay optimization protocol

The hemodynamic optimization protocol used a number of precise steps as previously described to increase the reliability of results.^6^, ^7^, ^8^, ^9^, ^10^, ^24^ In brief, patients in sinus rhythm had a range of AV delays tested from 40 to 320 ms in 40 ms increments until breakthrough of intrinsic conduction occurred. During the protocol, non‐invasive beat‐by‐beat BP was recorded (Finapres Medical Systems, Amsterdam, Netherlands). This is a digital photoplethysmograph that utilizes a volume‐clamp circuit via an inflatable cuff on the patient's index finger, to dynamically determine an arterial pressure waveform.^25^, ^26^ Simultaneously, a noninvasive laser Doppler sensor (Periflux 5000, Perimed, Järfälla, Sweden) recorded signals using a 780 nm wavelength laser, from the same beats, from the chest wall.

**FIGURE 1:**
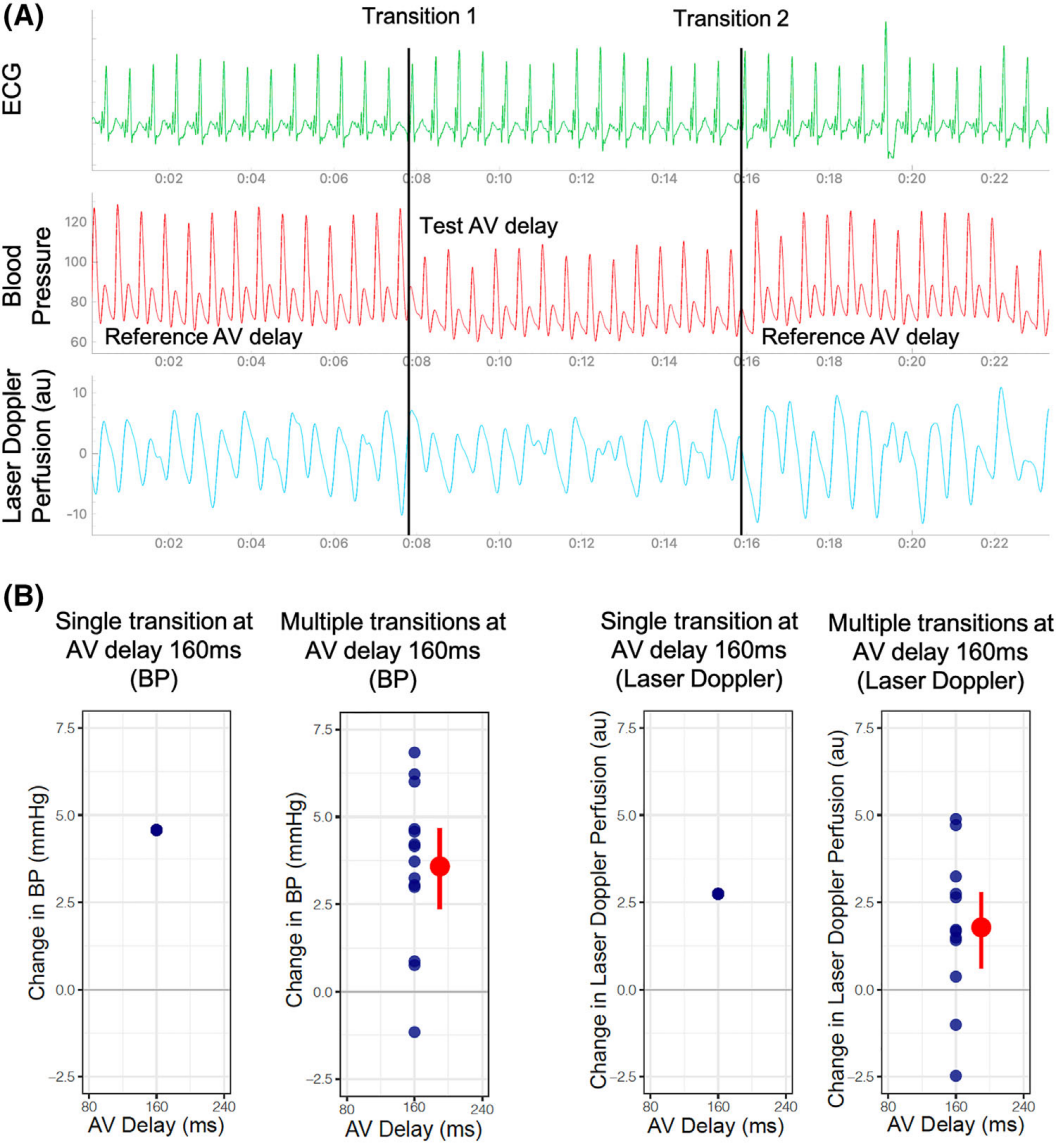
Simplified atrioventricular (AV) delay optimization protocol [Colour figure can be viewed at wileyonlinelibrary.com] We measured continuous noninvasive beat‐by‐beat blood pressure (BP) and noninvasive laser Doppler perfusion during our AV delay optimization protocol. (a) A minimum of 6 transitions were carried out between a tested AV delay (e.g., 80 ms) and a reference AV delay (120 ms). This is repeated at a range of AV delays from 40 to 320 ms until intrinsic conduction occurred. These changes are reflected in both the BP and laser Doppler traces. (b) At each alternation, a relative change in BP and laser Doppler perfusion are calculated. The mean relative change is then calculated from the average of multiple transitions and the standard error of the mean is plotted for each. This is repeated for each tested AV delay

Each tested AV delay (40, 80, 120, 160, 200, 240, 280, and 320 ms) was alternated with a reference state, either dual chamber pacing with an AV delay of 120 ms or atrial‐only pacing. For each patient the reference setting was the same throughout. This allowed a relative change in systolic BP or perfusion units to be calculated, respectively. This was the difference of the mean of eight beats prior to the transition compared with the mean of the eight beats that immediately follow. At each AV delay, transitions were repeated a minimum of six times. The standard error of the mean showed the precision of these hemodynamic changes. The protocol is shown in Figure 1.

For each individual optimization, a curve was fitted to the change in BP or change in laser Doppler.

The optimal AV delay was defined as the peak of the curve. Each optimization was carried out at a single fixed heart rate with the only change being the tested AV delay. Fusion was manually recorded at the time of data collection as well as during the processing of results based on ECG traces.

The analysis process was designed to minimize risk of bias through unblinded post‐processing of results. There was no manual editing of data, even to remove episodes containing ectopics. Future device‐based algorithms might build in systems to only conduct these optimizations when ectopics are relatively infrequent and to automatically exclude data containing ectopics. However, ectopics are frequent in patients with heart failure and there will be a trade‐off between demanding freedom from ectopics and demanding large quantities of data, both of which are desirable for precision. Our protocol handled this by favoring large quantities of data irrespective of the presence of ectopics and only rejecting the data if the automatic quality control system indicated that resulting optimum was imprecise. The protocol has the advantage for research purposes that it can collect data in finite time and that data would yield identical results if analyzed in this way by other researchers.

In a subset of patients, we tested serial AV delays against the reference state at both higher and lower rates to calculate the optimal laser Doppler perfusion and BP derived AV delays. This was used to assess the effect of higher rates on optimal AV delays and the quality control formula.

Separately, in another subset of patients, we tested an extended protocol that could be performed by an automated device algorithm to explore the impact of numbers of replicates (e.g., one replicate is a transition between the reference state and the tested AV delay) far beyond typical experimental protocols. We recorded 50, rather than the standard protocol, replicates of each transition. We then used bootstrapping analysis of 30 samples repeated 50 times, which provided a total of 1500 samples.

### 2.3 Automatic quality control algorithm

**FIGURE 2:**
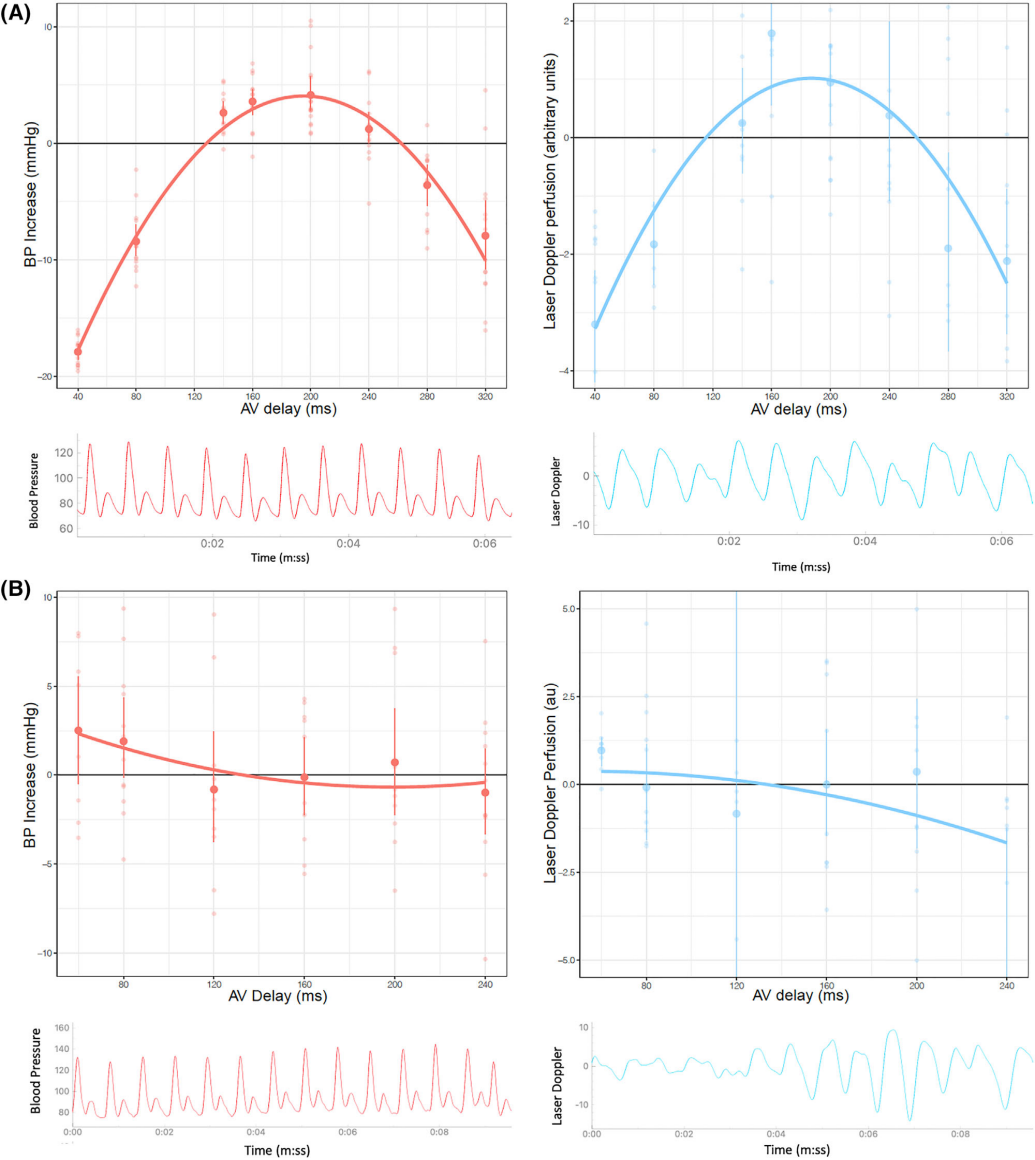
Examples of parabolas obtained following the atrioventricular (AV) delay optimization protocol for BP (left) and laser Doppler (right) measurements with examples of their corresponding signals (bottom panel). Figure 2a shows a clearly defined optimal AV delay and its corresponding good quality laser signal. Figure 2b shows no clear optimum with the corresponding poor quality (noisy) laser signal in spite of filtering. Note that when noise is large (2b), the orientations of the resulting parabolas are random, unlike the situation when noise is much smaller than the biological signal (2a) [Colour figure can be viewed at wileyonlinelibrary.com]

Because AV optima arise from the trade‐off between two undesirable processes (E wave truncation and A wave truncation), each of which have a curved relationship between AV delay degree of harm, the net effect on the final common pathway (such as cardiac output) is generally a curve. To a first approximation, this curve is a parabola with a maximum in the center decaying away to both sides. When fitting a parabola to multiple data points, the uncertainty in the position of the optimum can be calculated from the individual data points.^27^ This uncertainty, the standard error of the optimum (SE_OPT_), is informative because it is small when the points all lie in a convincing parabola (Figure 2A) and large when they do not, as happens when data is noisy (Figure 2B), or the parabola is relatively flat.

Our algorithm implements a two‐step quality control. First, it tests that the orientation of the optimization curve is correct, that is, an inverted U‐shape rather than a U‐shape. U‐shapes invariably represent noisy data. Second, it calculates the SE_OPT_ which is the uncertainty in the calculated AV optimum, and rejects optimizations with an excessively wide SE_OPT_, above 100 ms.

### 2.4 Statistical analysis

Data were processed using custom automated software in Python *(Python Foundation, Wilmington, Delaware, USA)*. Statistical analyses were performed on R version 3.6.2 *(R Project, Vienna, Austria)*. Signed differences between AV delays derived from laser Doppler and BP were quantified using the median difference and tested for being different from 0 using the Wilcoxon test. The variability of the differences was quantified using the median absolute deviation (i.e., the median of the absolute differences centered around the median difference). Because a few outlier points made the differences non‐Normally distributed, further analysis using the methods of Bland and Altman^28^, ^29^, ^30^ including the median bias are presented in the appendix (S4‐S6 Figures). The variability between the two methods is also presented as the interquartile range to allow the variability to be compared under different scenarios.

### 2.5 Study conduct

The studies from which we acquired the datasets were approved by local research ethics committees.^22^, ^23^ All patients gave written informed consent.

## 3 RESULTS

The study population consisted of 58 patients, 44 from the BRAVO trial and 14 from the HOPE‐HF trial. In 21 patients, multiple AV delay optimizations were performed at different heart rates. In total, across the 58 patients, 94 simultaneous laser Doppler and BP derived AV delay optimizations were performed. The mean patient age was 67.8 years, and 71% of patients were male. The other baseline characteristics are detailed in Table 1.

**TABLE 1:** Baseline characteristics

Baseline characteristics of patients (*n* = 58)	
Age, years**^a^**	68 (IQR: 62–76)
Male^†^	41 (71%)
NYHA functional class^b^	
Class I	0 (0%)
Class II	48 (83%)
Class III	10 (17%)
Class IV	0 (0%)
CRT lead type†^b^	
LV lead	44 (76%)
His bundle lead	14 (24%)

**Abbreviations**: CRT, cardiac resynchronization therapy; IQR, inter‐quartile range; LV, Left ventricular; NYHA, New York Heart Association.

^a^ Median (IQR).

^b^ *n* (%).

### 3.1 Shape of hemodynamic response

Of the 94 laser Doppler optimizations, 79 (84%) curves were appropriately orientated (p < .0001) and had a median SE_OPT_ of 53 ms (IQR 37–82 ms). Of the 94 BP optimizations, 90 (96%) curves were appropriately orientated and had a median SE_OPT_ of 26 ms (IQR 15–46 ms).

### 3.2 Primary results: Comparison of laser Doppler and BP derived optima

**FIGURE 3:**
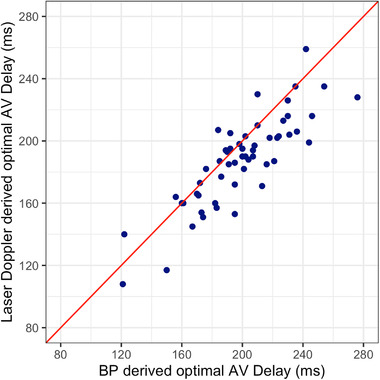
Association between optimal AV delays found using BP and laser Doppler. As expected, there is a significant correlation between the laser Doppler and BP derived optimal AV delays (Spearman's rho 0.82, p < .0001) [Colour figure can be viewed at wileyonlinelibrary.com]

Of the 94 optimization sessions, in 76 both the laser Doppler and BP curves had the physiologically appropriate orientation. In only 55 sessions, did both curves also meet the second requirement, SE_OPT _< 100 ms (Figure 3). The median difference between the laser and BP derived AV delay was −12 ms (median absolute deviation 12 ms).

Among the 74 optimization sessions in patients with biventricular pacing, 49 had both the appropriate orientation and an SE_OPT_ < 100 ms. The median difference between the laser Doppler and BP derived AV delay was −10 ms (median absolute deviation 11 ms).

Among the 18 optimization sessions in patients with his bundle pacing, six had laser Doppler and BP curves in the appropriate orientation and an SE_OPT _< 100 ms. The median difference between the laser Doppler and BP derived AV delay was −22.5 ms (median absolute deviation 8.5 ms).

### 3.3 The effect of heart rate on the agreement between laser Doppler and BP derived AV delays

Of the 21 patients who underwent optimization at two heart rates, 13 passed the quality control step at both heart rates for both laser Doppler and BP.

The AV optima showed a tendency to be a little shorter at higher heart rates than at lower heart rates:

by laser Doppler 189 versus 193 ms, p = .46; by BP 190 versus 206 ms, p = .01. The uncertainty of the optimum (SE_OPT_) showed a tendency to be smaller (i.e., better) at higher heart rates: by Laser Doppler 46 versus 53 ms, p = .3; BP 13 ms versus 21 ms, p = .006.

### 3.4 Effect of number of replicates on precision of optimization

We noticed that many of the optimizations were failing the quality control criteria, that is, had large enough noise to either make the best fit curve upside down or to leave a large uncertainty in the position of the optimum.

We therefore conducted an additional experiment on five further patients who underwent a very extended protocol of 50 replicates, rather than 6 to 10, to test our assumption that a more extended protocol would reduce the uncertainty of the optimization (reduce the SE_OPT_).

**FIGURE 4:**
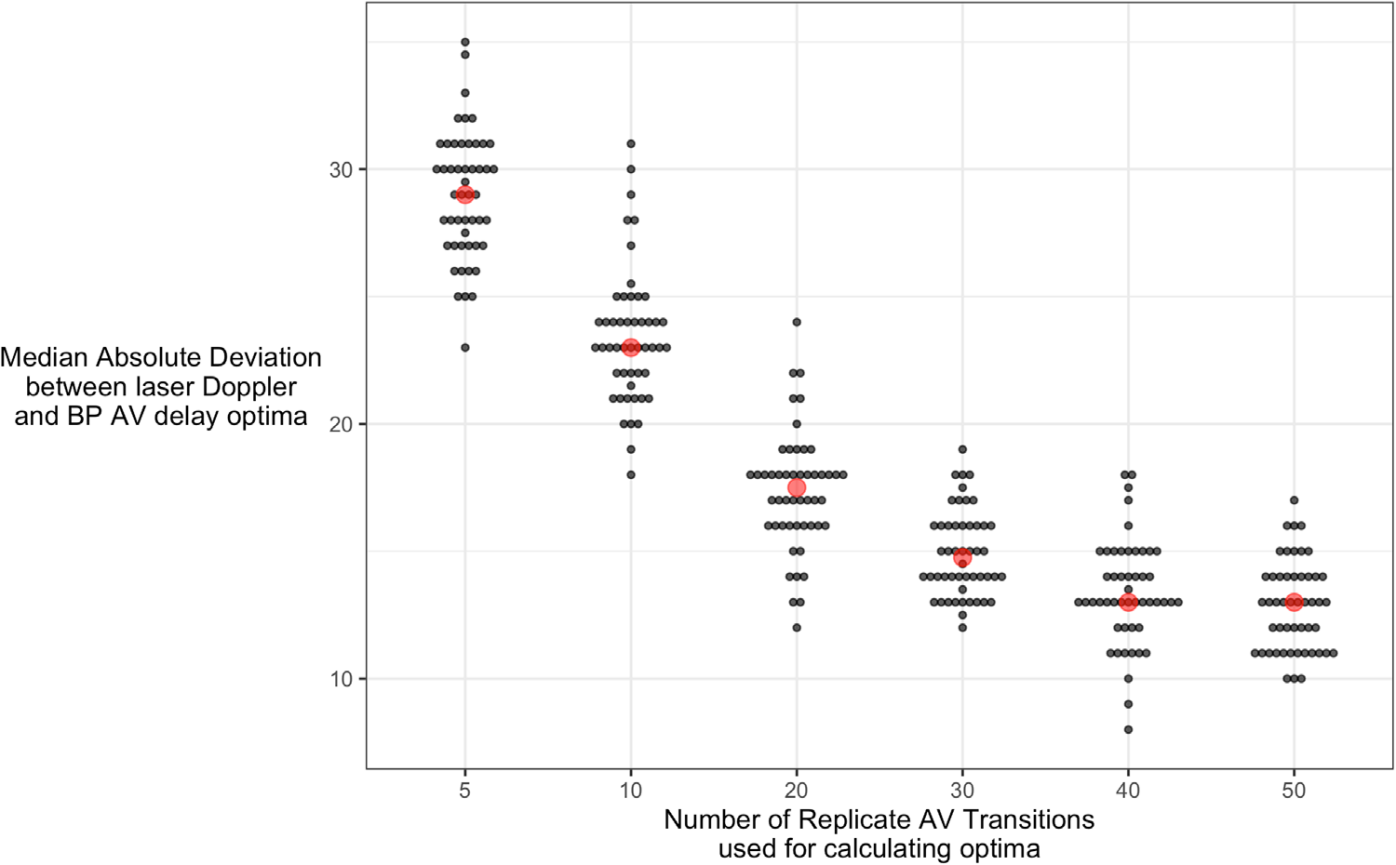
Effect of number of replicates in the protocol on the agreement between laser Doppler and blood pressure optima. If we trim the optimization session data to just five replicates per AV delay transition, there is considerable disagreement between laser Doppler and blood pressure derived AV delay optima (black dots in left column with median shown as red dot). These results arise from 30‐fold bootstrapping analysis of the extended‐protocol patients. As we use progressively more of the data from each optimization session, the disagreement in AV delay optima between laser Doppler and blood pressure progressively falls [Colour figure can be viewed at wileyonlinelibrary.com]

Within these five extended‐protocol patients, we could therefore test replicate counts from 5 to 50, assessing the mismatch between laser Doppler optimum and BP optimum for each replicate count. With only five replicates, the disagreement between the optima (expressed as a median absolute deviation) was significantly wider than with our standard 6 to 10 replicate protocol (29 vs. 23 ms, p < .0001). With 50 replicates, the disagreement was significantly smaller (13 vs. 23 ms, p < .0001). Figure 4 shows the progressive decline in disagreement between laser Doppler and BP optima as the number of replicates in the optimization protocol is increased (p < .0001 for trend).

## 4 DISCUSSION

In this study we found that laser Doppler derived AV delay optimizations can be clinically equivalent to those derived from BP measurements with three specific findings. First, a laser Doppler optimum meeting automated quality control criteria can be trusted to be clinically representative of the BP optimum. Second, about a third of optimization sessions consisting of 6 to 10 replicates per AV delay, fail the quality control criteria. Third, increasing the number of replicates powerfully reduces the uncertainty of the optimum, manifesting in passing the quality control.

### 4.1 Clinical implications

Laser Doppler perfusion monitoring has been miniaturized and can be incorporated into devices. This allows an accurate set of measurements to be performed frequently without any inconvenience to the patient or the healthcare system. In particular, because what is measured is downstream from the heart, it is the end result of the complex physiology within the heart and can be used without the need for expert acquisition and interpretation of intra‐cardiac Doppler waveform.

Implantation of this technology may also assist devices to make a physiologically based decision on whether to deliver therapy in suspected tachycardias. We have previously found that the addition of perfusion data successfully improves discrimination of when therapy is appropriate.^20^

### 4.2 Importance of numbers of replicates

Performing multiple replicates of each transition has two benefits. First, the precision of the estimates at each AV delay is improved by the averaging process, reducing the impact of the intercurrent biological fluctuation. Our primary experimental protocol was just 6 to 10 replicates because we had found this to be sufficient in the past for BP based optimizations.^8^, ^12^ However, this study revealed that laser Doppler gives a noisier signal than BP, and therefore more replicates are required to achieve the same precision.

This study is the first to document the progressive decline in disagreement between laser Doppler and BP derived AV delay optima as the number of replicates is increased. This arose from the extended protocol, which we asked five patients to undergo, after we had analyzed the results of the main study. The extended results confirmed the expectation of the precision increasing progressively as the number of replicate transitions in the protocol are increased.

In the ultimate clinical application of an automatic optimization system such as this, the patient does not have to attend for a session and therefore long duration is not a burden. Moreover, data from separate sessions can be merged during curve fitting. In an extreme example, a patient could undergo a single alternation per AV delay today, another tomorrow, and so on, and after 50 days, the device could construct a parabola using the full data. We have previously found that hemodynamic optima are stable for many months.^31^

### 4.3 Automatic quality control

The second reason to perform multiple replicates is that the variation between the replicates provides crucial information on the uncertainty in the resulting AV optimum calculation. It is essential to check this uncertainty rather than just accept the highest value as the optimum because if the SE_OPT_ is wide, in most cases, the highest recorded BP will not be at the true hemodynamic optimum.^27^, ^32^, ^33^

An inverted parabola is a clear sign that the noise has swamped the signal and can easily be rejected. However, when noise swamps the signal, in only half the cases will the resulting parabola be inverted. In the other half, the parabola will be upright and mistaken for a genuine optimum if quality control does not include calculation of SE_OPT_.

### 4.4 Underlying concordance of laser Doppler and BP optima

The optima of laser Doppler mirror those of BP. They are not identical, but this is inevitable because even two repeated BP optimizations or two repeated laser Doppler optimizations will not be identical, which puts a floor on the agreement between the two methods. A key finding from this study is that there is no consistent tendency for the laser Doppler optimum to be longer or shorter by a clinically significant amount than those of BP.

The SE_OPT_ of laser Doppler is, however, wider than that of BP, by a factor of approximately two. This means that to have the same precision as a BP optimization, the laser Doppler optimization would need more replicates. Whilst this may be impractical in an experimental laboratory setting where the patient has to attend specially and staff has to perform the procedure, it is not a significant limitation for an automated optimization algorithm that could run autonomously inside a device, as would be possible with an implanted laser Doppler sensor.

### 4.5 Study limitations

Our main protocol used only the modest number of replicates (6–10) that we had found suitable for BP‐based optimization in the past, because it was carried out on a subset of patients who were anyway undergoing BP‐based optimization as part of the HOPE‐HF and BRAVO trials.^22^, ^23^ Only after analyzing the data from the main protocol, was it clear to us that more replicates would be needed for laser Doppler optima to achieve that level of precision. The small extended protocol cohort verified the increase in precision with greater number of AV transition replicates. Reduction in SE_OPT_ is routinely expected from the underlying mathematics of curve fitting.^27^

Ours is the first study to report SE_OPT_ on a large group of patients. With the 6 to 10 replicate cycles of each AV delay that was practical in an outpatient laboratory setting, the SE_OPT_ was still rather wide. To obtain optima more precisely than this, all that is required is more replicates for each AV delay for each patient. Roughly speaking, SE_OPT_ is halved when the number of replicates is quadrupled. In HOPE‐HF and BRAVO, the hundreds of changes of pacemaker settings that each patient required had to be performed manually, while the patient waited in the laboratory.

However, the ultimate application of this technology would be an autonomous protocol delivered by the device itself, carried out without inconvenience to the patient whilst they were at home. This would allow two advantages. First, there could be many more replicates, so the precision of optimization would immediately be increased. The extended experiments in five patients and associated bootstrapping analysis demonstrate the clear progressive reduction in SE_OPT_ with increase in number of replicates that would be expected from first principles: this decline should continue as the number of replicates increase even further. A second potential advantage is that each replicate could be longer, allowing more time of recording of the slightly later period after the transition where our previous experiments indicate flow signals might be even larger.

We did not test other light‐based sensors and therefore, have not assessed if alternative sensors would provide an improved signal‐to‐noise ratio. However, we have shown that laser Doppler has an acceptable signal‐to‐noise ratio with an appropriate protocol.

Further research is still required before laser Doppler can be incorporated into a device. Chronic safety and signal fidelity of laser Doppler implantation needs to be assessed, expanding on pilot recordings using a miniaturized laser Doppler prototype.^20^, ^34^, ^35^

## 5 CONCLUSION

Optimal AV delays derived from noninvasive beat‐by‐beat BP or laser Doppler methods are clinically equivalent, although the automated quality control criteria highlight that the laser Doppler signals are individually more noisy than BP signals and therefore require more replicate transitions to achieve the same precision. Precision of the optimization can be verified automatically from within the acquired data itself, so that the number of replicates can be tuned as required. Since laser Doppler sensors are more suitable for miniaturization and implantation than BP sensors, coupled with an automatic quality control algorithm they may enable future cardiac devices to dynamically and reliably optimize AV delays.

## AUTHOR CONTRIBUTIONS

Conceived and designed the experiments: Zachary I Whinnett, Darrel P Franics, Daniel Keene.

Performed the experiments: Zachary I Whinnett, Daniel Keene, Ahran D Arnold, Alejandra A Miyazawa, Nadine Ali, Khulat A Saqi, Leah J Burden, Katherin March.

Statistical Analysis: Alejandra A Miyazawa, Monika Johal, Matthew J Shun‐Shin.

Drafted the initial manuscript: Alejandra A Miyazawa, Daniel Keene, Monika Johal, Matthew J Shun‐Shin.

Critically revised manuscript: Alejandra A Miyazawa, Daniel Keene, Darrel P Franics, Zachary I Whinnett, Matthew J Shun‐Shin.

Final Approval: Daniel Keene, Alejandra A Miyazawa, Monika Johal, Ahran D Arnold, Nadine Ali, Khulat A Saqi, March, Leah Burden, Darrel P Franics, Zachary I Whinnett, Matthew J Shun‐Shin.

## Supporting information


**Table S1**. Inclusion and exclusion criteria
**Figure S1**. Laser Doppler Perfusion Monitoring
**Figure S2**. Experimental protocol set up
**Figure S3**. Quality control formulas derived by Francis DP
**Figure S4**. Bland‐Altman plot of the differences between LDPM and BP derived atrioventricular delays with no quality control applied (n = 94)
**Figure S5**. Bland‐Altman Plots (left) and Scatter Plots (right) of the differences in laser Doppler and BP (running mean) derived atrioventricular (AV) delays with the quality control formula
**Figure S6**. Bland‐Altman Plots (Left) and Scatter Plots (Right) of the differences in laser Doppler and BP derived atrioventricular delays at different heart rates without a quality control
