# Supplementary material for: Optimizing atrio‐ventricular delay in pacemakers using potentially implantable physiological biomarkers
Source: Pacing Clin Electrophysiol. 2022 Jan 28;45(4):461–70. doi: 10.1111/pace.14434 (PMC9305784; doi:10.1111/pace.14434)
Supplement: Supplementary file 1 — Table S1. Inclusion and exclusion criteria Figure S1. Laser Doppler Perfusion Monitoring Figure S2. Experimental protocol set up Figure S3. Quality control formulas derived by Francis DP Figure S4. Bland‐Altman plot of the differences between LDPM and BP derived atrioventricular delays with no quality control applied (n = 94) Figure S5. Bland‐Altman Plots (left) and Scatter Plots (right) of the differences in laser Doppler and BP (running mean) derived atrioventricular (AV) delays with the quality control formula Figure S6. Bland‐Altman Plots (Left) and Scatter Plots (Right) of the differences in laser Doppler and BP derived atrioventricular delays at different heart rates without a quality control [file PACE-45-461-s001.docx]

**Supplementary Materials**

| \| **BRAVO Trial** \| \| \| --- \| --- \| \| **Inclusion Criteria** \| **Exclusion Criteria** \| \| Previous diagnosis of chronic heart failure \| Major cardiovascular event within 6 weeks of enrolment \| \| CRT device implanted at least 6 months before enrolment \| Uncontrolled hypertension \| \| History of symptomatic congestive heart failure (NYHA functional class II-IV) \| Inability to walk on a treadmill \| \| Prior ejection fraction < 40% or documented moderate to severely impaired systolic function \|  \| \| Stable medical therapy for heart failure \|  \| \| >90% biventricular pacing \|  \|  \| **HOPE-HF Trial** \| \| \| --- \| --- \| \| **Inclusion Criteria** \| **Exclusion Criteria** \| \| Age over 18 years \| Permanent or persistent AF \| \| EF ≤ 35% or EF 36-40% with BNP >250 pg/mL \| Paroxysmal AF with history of sustained AF (24h) in the 6 months prior to screening \| \| NYHA functional class II-IV \| Lack of capacity to consent \| \| Sinus Rhythm \| Other serious medical condition with life expectancy of <1 year \| \| PR interval ≥ 200ms \| Pregnancy \| \| QRS duration ≤ 40ms or prolonged QRS duration with typical RBBB morphology \|  \| |
| --- | --- | --- | --- | --- | --- | --- | --- | --- | --- | --- | --- | --- | --- | --- | --- | --- | --- | --- | --- | --- | --- | --- | --- | --- | --- | --- | --- | --- | --- | --- | --- | --- |
| **Table S1. Inclusion and exclusion criteria**  The inclusion and exclusion criteria for patients involved in the BRAVO trial are shown in the top panel.  The inclusion and exclusion criteria for patient’s involved in the HOPE-HF trial are shown in the bottom panel.  NYHA = New York Heart Association classification. AF = atrial fibrillation. RBBB = right bundle branch block. Table information was obtained from the BRAVO trial and HOPE-HF trial respectively (22,23). |

| 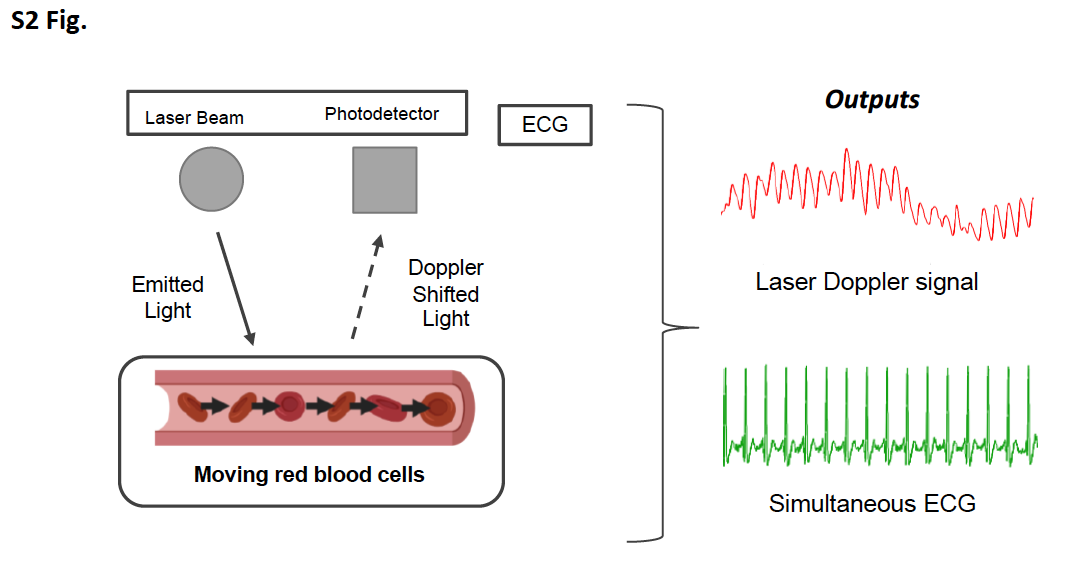 |
| --- |
| **Figure S1. Laser Doppler Perfusion Monitoring**  This demonstrates how laser Doppler perfusion monitoring works by quantifying blood flow based on the doppler shift of transmitted light by moving red blood cells and producing an oscillatory trace. The oscillations are synchronous with an R wave from a simultaneously recorded ECG trace. Figure produced based on previously published figures using ©BioRENDER.  ECG: electrocardiogram |

| **** |
| --- |
| **Figure S2. Experimental protocol set up**  Visual representation of ECG lead positions, laser doppler probe and non-invasive blood pressure monitoring via the Finometer during the experimental protocol.  Laser Doppler signals are transmitted to the Perimed Periflux 5000 device via a fibre optic cable. They are then converted from analogue to digital signals alongside simultaneous inputs from the ECG and Finometer. This information is then stored on a computer for offline analysis. (22,23)  ECG: electrocardiogram; SBP: systolic blood pressure |

| **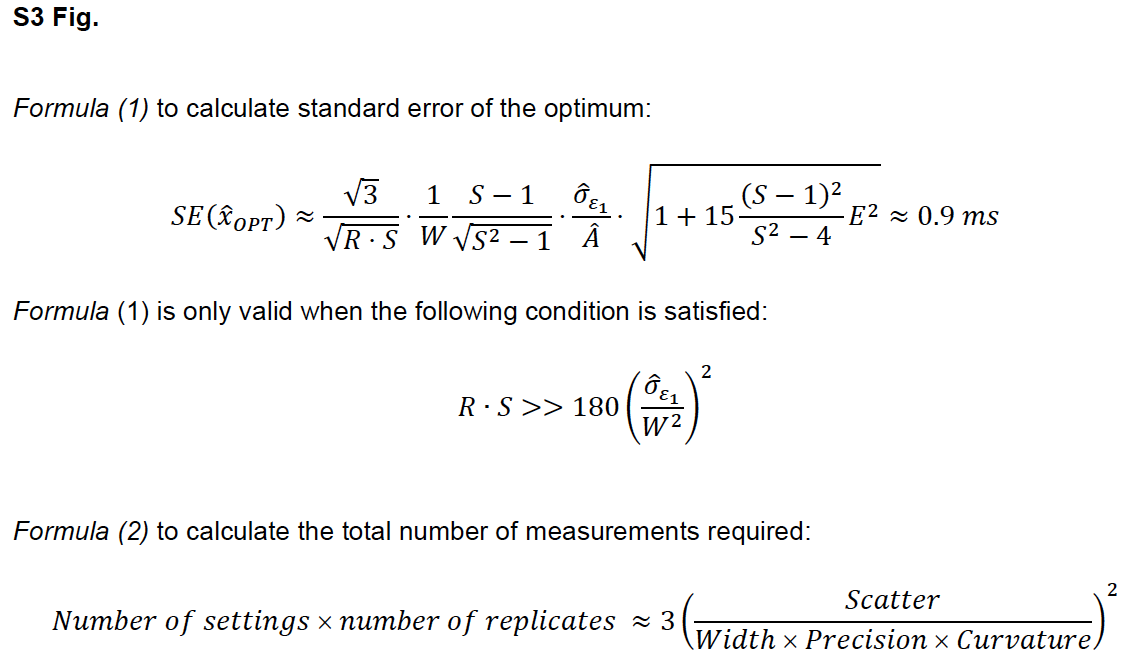** |
| --- |
| **Figure S3. Quality control formulas derived by Francis DP**  Formulas as referenced in the flowchart (Fig 7). Use of these formulas is restricted to atrioventricular delays obtained by fitting a quadratic curve. Formula (1) can be applied to calculate the standard error of the optimum. This formula is only valid when the condition shown below formula (1) is satisfied as this ensures that the bare minimum number of raw measurements required to validly estimate the standard error of the optimum have been taken. Formula (2) can be applied in-clinic to calculate the total number of settings and replicates required.  $\mathrm{SE}\left( \hat{x}_{\mathrm{OPT}} \right)$= Standard error of the optimum AV delay  S = Settings, is the number of tested AV delays e.g. 5  R = Replicates, is the number of multiple replicate measurements performed at each AV DELAY e.g. 3  W = Width (ms) is the tested range of AV delays e.g. 120ms  $\hat{A}$= Curvature (physiological units of response per ms^2^), is described by the coefficient of χ^2^ e.g. 0.0083mmHg ms^-2^  $\hat{\sigma}_{\varepsilon_{1}}$= Scatter, is the standard deviation of multiple replicate measurements performed at one AV delay e.g. 2.5mmHg  E = Extremeness, how far out the optimum is from the middle of the tested range e.g. 0.2  The precision (ms) can be set by clinicians according to the desired standard error of the optimum.  Figure produced based on previously published formulas (27). |

| 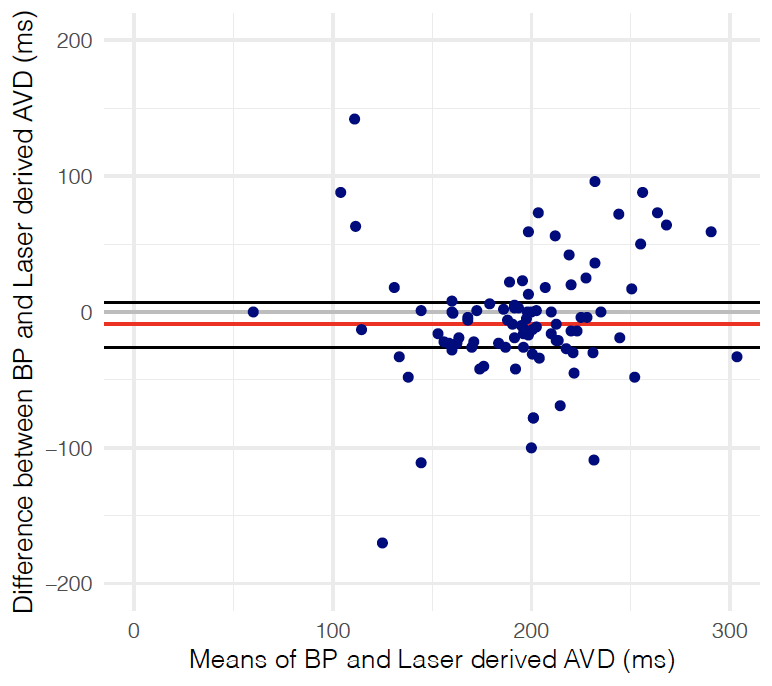 |
| --- |
| **Figure S4.** **Bland-Altman plot of the differences between LDPM and BP derived atrioventricular delays with no quality control applied (n=94).**  The red horizontal line represents the median bias between the two measurement methods and the black lines represent the interquartile range. |

| 1. SE_OPT_ <50ms   **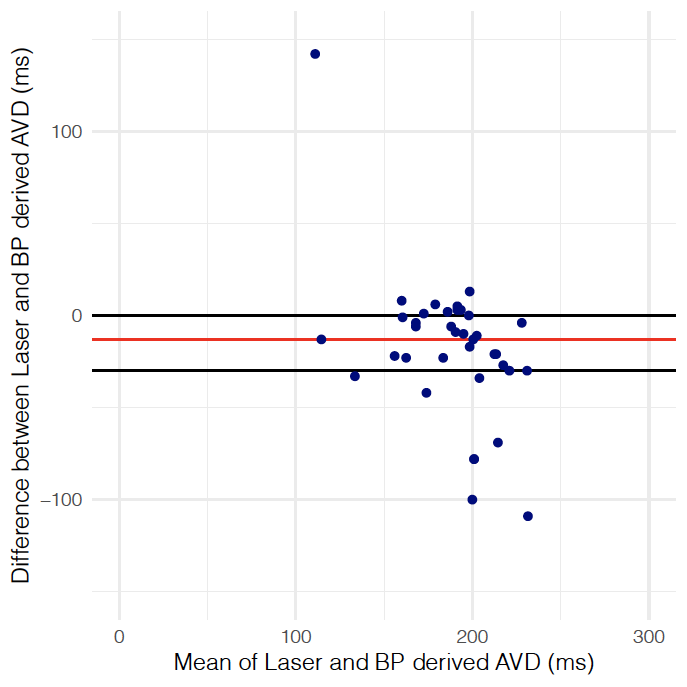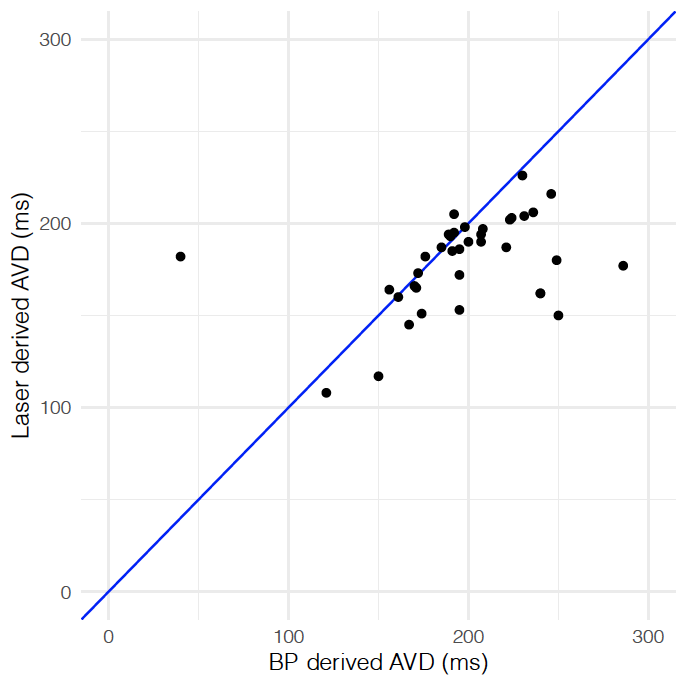**   1. SE_OPT_ <75ms   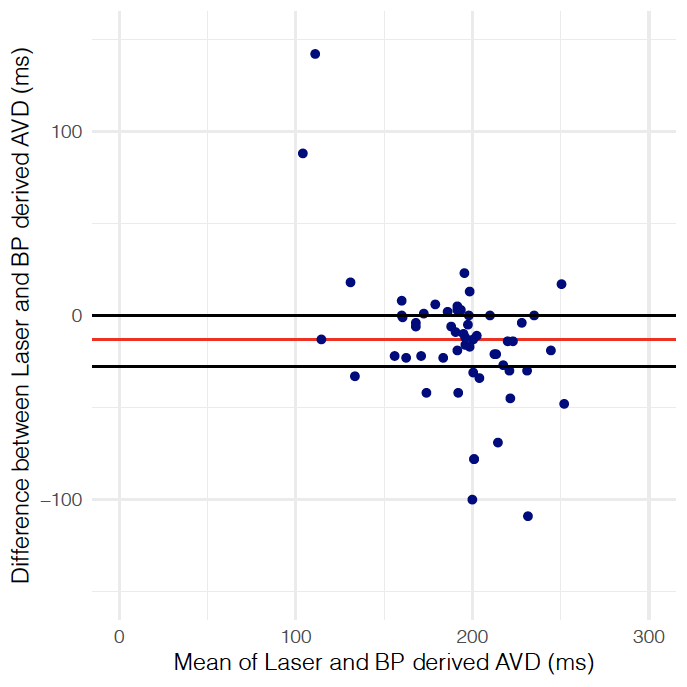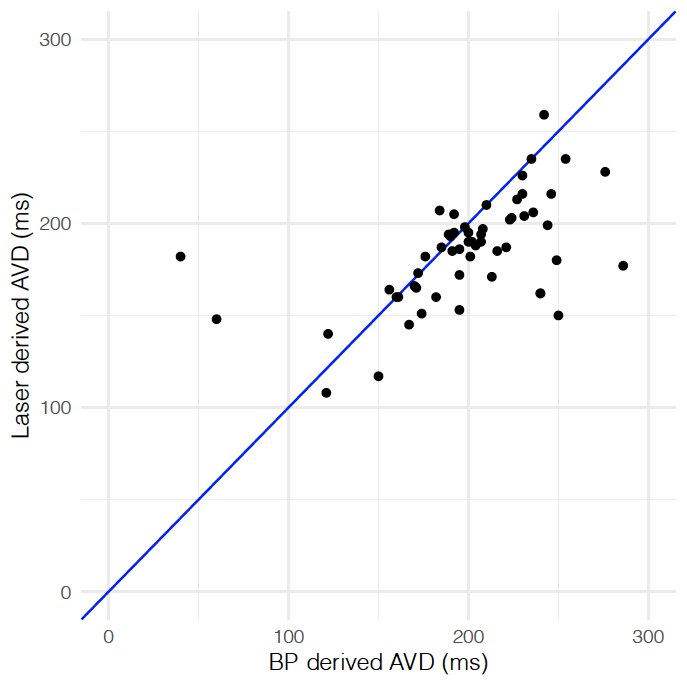   1. SE_OPT_ <100ms   **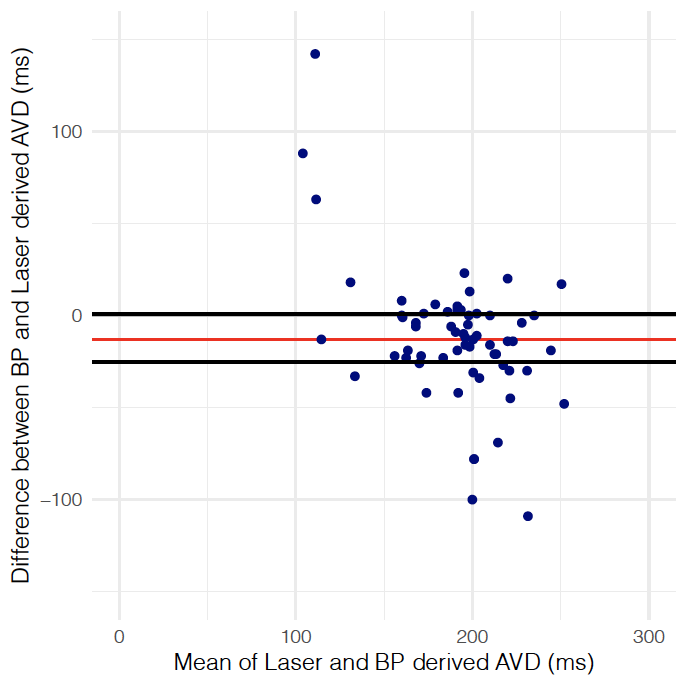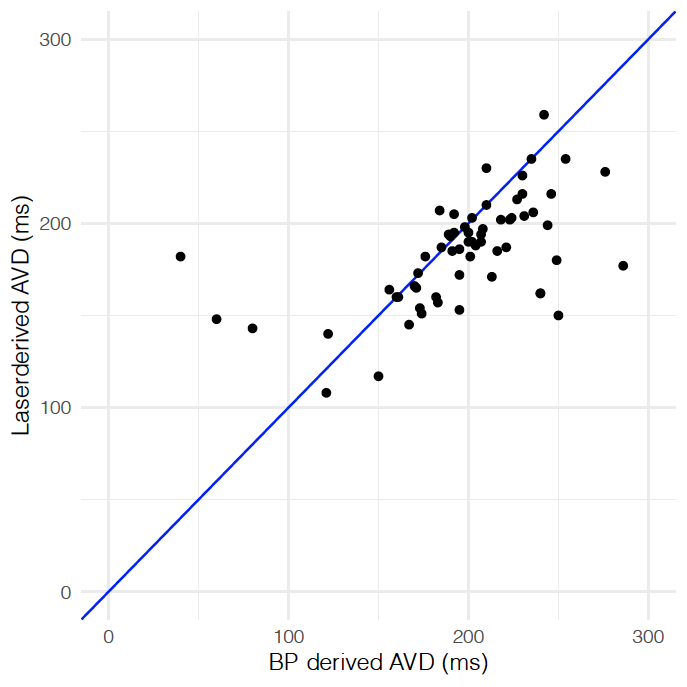** |
| --- |
| **Figure S5.** **Bland-Altman Plots (left) and Scatter Plots (right) of the differences in laser Doppler and BP (running mean) derived atrioventricular (AV) delays with the quality control formula.**(41)  In Fig S5, we use the quality control formula and include data sets with a standard error of their optimum (SE_OPT_) <50ms (5a), <75ms (5b) and <100ms (5c). For Bland-Altman plots, the red horizontal line represents the median bias between the two measurement methods and the black lines represents the interquartile range (25^th^ and 75^th^ percentile).  For scatter plots, the blue line represents the line of equality.  AV: atrioventricular  AVD: atrioventricular delay  BP: Blood pressure |

| **Figure S6a. Differences in laser Doppler and BP derived AV delays at higher heart rates**  **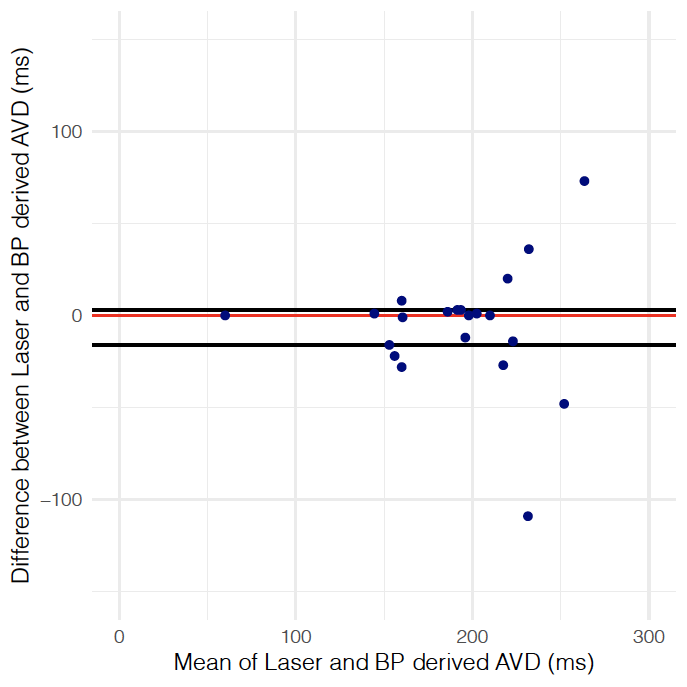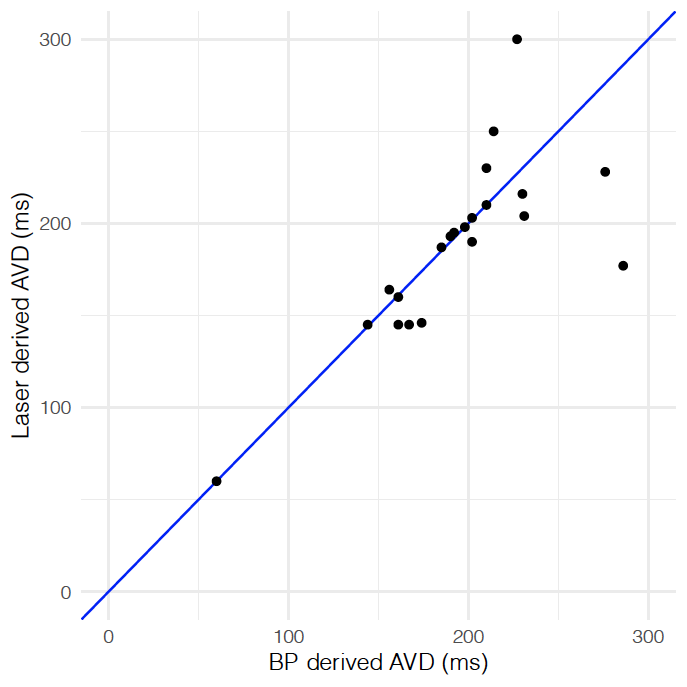**  **Figure S6b. Differences in laser Doppler and BP derived AV delays at lower heart rates**  **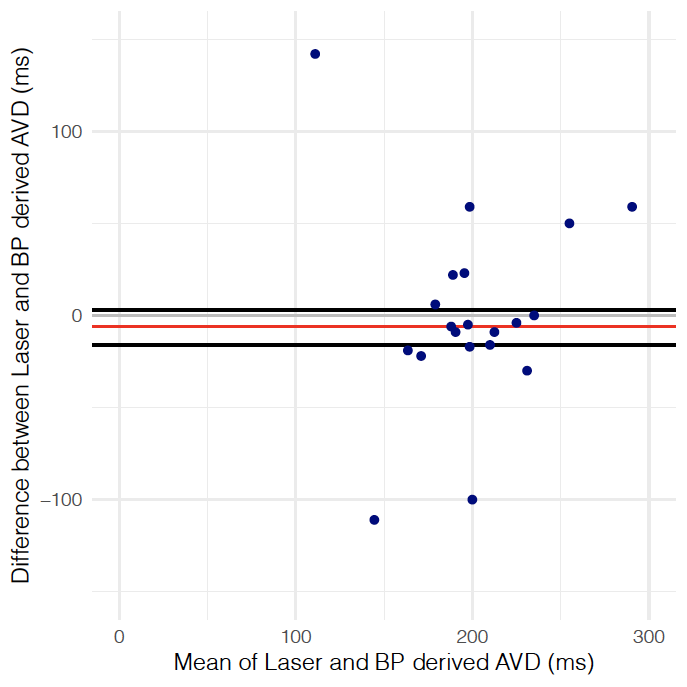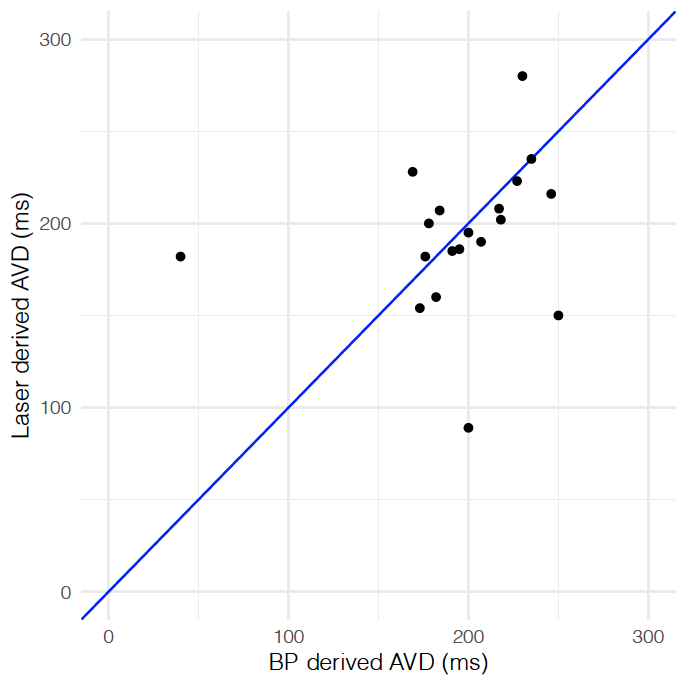** |
| --- |
| **Figure S6. Bland-Altman Plots (Left) and Scatter Plots (Right) of the differences in laser Doppler and BP derived atrioventricular delays at different heart rates without a quality control.**  21 patients had their AV delays tested at both a higher heart rates (Figure 6a) and lower heart rates (Figure 6b). For Bland-Altman plots, the red horizontal line represents the median bias between the two methods and the black lines represent the interquartile range (25^th^ and 75^th^ percentile).  For scatter plots, the blue line represents the line of equality.  AVD: Atrioventricular delay  AV: atrioventricular  BP: blood pressure |
